# Diseases of the Skin and the Eruptive Fevers

**Published:** 1909-07

**Authors:** 


**Diseases of the Skin and the Eruptive Fevers.** By **Jay Frank Schamberg,** M.D., Professor of Dermatology and Infectious Eruptive Diseases in the Philadelphia Polyclinic and College for Graduates in Medicine. Octavo of 534 pages, illustrated. Philadelphia and London: W. B. Saunders Company, 1908. (Cloth, $3.00 net.)

The author of this admirable volume has succeeded in condensing an astonishing amount of information into a comparatively small compass. The work is essentially clinical and practical. It is divided into two chapters, the first being devoted to diseases of the skin, the descriptions of which include the various dermatoses. The book is strongly recommended to students who, while they may not have time, or inclination to consult the large treatises on skin diseases, nevertheless desire a general and practical knowledge of the subject.

A very important and most useful feature of the work consists of the photographic reproductions. Some of these are exceptionally fine and the student will find them a great help in his researches.

The work differs from others in that the exanthemata are treated in a separate chapter, and have more space allotted to them than is given in any work of equal size relating to dermatology. This is well, considering the many errors from lack of training committed by the average practitioner, and the harm that may follow: for instance, the diagnosis that mistakes smallpox for "Cuban itch," a term employed as a mere convenience. The reviewer once visited a town in which existed two hundred cases of smallpox, which each one of the seven local physicians had diagnosed as that purely fanciful disease, a striking commentary upon the meagre training given in our universities to the important matter of general dermatology. The time is ripe for emphasising the close relation that the diagnosis of the exanthemata has to various dermatoses, which is here admirably done. Recognising the necessity of a thorough understanding of the various failures due to physical symptoms, in order that the facts elicited may be properly interpreted the author has spared no pains in showing their basal and underlying principles.

